# The Impact of Aging on the Association Between Aortic Stiffness and Cerebral Pulsatility Index

**DOI:** 10.3389/fcvm.2022.821151

**Published:** 2022-02-09

**Authors:** Brandon G. Fico, Kathleen B. Miller, Leonardo A. Rivera-Rivera, Adam T. Corkery, Andrew G. Pearson, Nicole A. Eisenmann, Anna J. Howery, Howard A. Rowley, Kevin M. Johnson, Sterling C. Johnson, Oliver Wieben, Jill N. Barnes

**Affiliations:** ^1^Bruno Balke Biodynamics Laboratory, Department of Kinesiology, University of Wisconsin-Madison, Madison, WI, United States; ^2^Wisconsin Alzheimer's Disease Research Center, School of Medicine and Public Health, University of Wisconsin-Madison, Madison, WI, United States; ^3^Department of Medical Physics, School of Medicine and Public Health, University of Wisconsin-Madison, Madison, WI, United States; ^4^Department of Radiology, School of Medicine and Public Health, University of Wisconsin-Madison, Madison, WI, United States; ^5^Geriatric Research Education and Clinical Center, William S. Middleton Memorial Veteran's Hospital, Madison, WI, United States

**Keywords:** pulse wave velocity, arterial stiffness, cerebrovasculature, cerebral hemodynamics, 4D flow MRI

## Abstract

The central arteries dampen the pulsatile forces from myocardial contraction, limiting the pulsatility that reaches the cerebral vasculature, although there are limited data on this relationship with aging in humans. The purpose of this study was to determine the association between aortic stiffness and cerebral artery pulsatility index in young and older adults. We hypothesized that cerebral pulsatility index would be associated with aortic stiffness in older adults, but not in young adults. We also hypothesized that both age and aortic stiffness would be significant predictors for cerebral pulsatility index. This study included 23 healthy older adults (aged 62 ± 6 years) and 33 healthy young adults (aged 25 ± 4 years). Aortic stiffness was measured using carotid-femoral pulse wave velocity (cfPWV), while cerebral artery pulsatility index in the internal carotid arteries (ICAs), middle cerebral arteries (MCAs), and basilar artery were assessed using 4D Flow MRI. Cerebral pulsatility index was calculated as (maximum flow – minimum flow) / mean flow. In the combined age group, there was a positive association between cfPWV and cerebral pulsatility index in the ICAs (*r* = 0.487; *p* < 0.001), MCAs (*r* = 0.393; *p* = 0.003), and basilar artery (*r* = 0.576; *p* < 0.001). In young adults, there were no associations between cfPWV and cerebral pulsatility index in any of the arteries of interest (ICAs: *r* = 0.253; *p* = 0.156, MCAs: *r* = −0.059; *p* = 0.743, basilar artery *r* = 0.171; *p* = 0.344). In contrast, in older adults there was a positive association between cfPWV and cerebral pulsatility index in the MCAs (*r* = 0.437; *p* = 0.037) and basilar artery (*r* = 0.500; *p* = 0.015). However, the relationship between cfPWV and cerebral pulsatility index in the ICAs of the older adults did not reach the threshold for significance (*r* = 0.375; *p* = 0.078). In conclusion, age and aortic stiffness are significant predictors of cerebral artery pulsatility index in healthy adults. This study highlights the importance of targeting aortic stiffness in our increasingly aging population to reduce the burden of age-related changes in cerebral hemodynamics.

## Introduction

As the lifespan of older adults continues to increase, the percentage of this subset of the population over 65 years of age is expanding, and is expected to surpass the amount of children in the United States for the first time by 2034 (1). The leading cause of death in adults 65 years and older is cardiovascular disease due to increases in the prevalence of atherosclerosis, hypertension, myocardial infarction, and stroke (2). Mechanistically, aortic stiffness increases with advancing age (3) and there is substantial evidence that it is predictive of cardiovascular disease and mortality (4). Specifically, a 1 m/s increase in carotid-femoral pulse wave velocity (cfPWV) is associated with a 7% increased risk of cardiovascular events (5). Age-related increases in aortic stiffness result in elevated pulse pressure and incident hypertension (6).

Due to arterial stiffening, aortic impedance increases with advancing age, while impedance in the distal muscular arteries does not increase, leading to greater impedance mismatching and resulting pulsatility (7, 8). Additionally, cerebrovascular impedance has also been demonstrated to increase with age (9). As such, this increased arterial stiffness with aging facilitates excessive pressure and flow pulsatility and may result in microvascular damage (10). Of concern, the brain is a high perfused organ and is susceptible to excessive pressure and increased pulsatility index (11). It has been demonstrated that increased aortic stiffness impairs memory via increased white matter hyperintensities and higher prevalence of subcortical infarctions (10). As such, cerebral artery pulsatility index has been demonstrated to be elevated in patients with vascular dementia (12, 13) and Alzheimer's disease (14–16), both of which increase in prevalence with advancing age. It has also been postulated that intracranial arteries are less compliant in patients with Alzheimer's disease, thereby increasing cerebral pulsatility index and exacerbating the pulsatile forces that reach the cerebral microvessels (15).

In animal models (e.g., mice) it has been demonstrated that increased arterial stiffness via imposed carotid artery calcification (typically observed with aging) results in increased blood flow pulsatility in the cerebral arteries (17), while studies in humans have described increased cerebral pulsatility as a consequence of elevated peripheral resistance (18). Additionally, cerebral pulsatility has also been linked to elevated arterial stiffness in middle-aged adults using transcranial doppler ultrasound (19). As such, we can assume that increased aortic stiffness with aging results in augmented cerebral vessel pulsatility index. However, the impact of age on the association between aortic stiffness and cerebral pulsatility index in multiple intracranial vessels has yet to be evaluated. Therefore, the purpose of this study was to compare the association of aortic stiffness (as measured by cfPWV) and cerebral pulsatility index in the internal carotid arteries (ICAs) middle cerebral arteries (MCAs), and basilar artery in young adults vs. older adults, in the absence of clinical disease. We hypothesized that cerebral pulsatility index in the older adults would be associated with cfPWV, but not in the young adults. We also hypothesized that both age and cfPWV would be significant predictors for cerebral pulsatility index.

## Methods

### Participants

Thirty-three young (between 18 and 35 years old) and twenty-three older (between 50 and 68 years old) physically active healthy adults participated in the study. Participants were excluded from the study if they had a body mass index (BMI) > 30 kg/m^2^, and if they 1) were current smokers; 2) were diagnosed with hypertension based on the latest guidelines (20) or taking blood pressure medications; 3) presented with a history or evidence of hepatic or renal disease, hematological disease, peripheral vascular disease, stroke, neurovascular disease, cardiovascular disease, diabetes; or 4) had contraindications for magnetic resonance imaging (MRI) scan (as determined by a health history questionnaire and MRI screening form). All scans were reviewed by a neuroradiologist (HAR) for incidental findings. Physical activity was determined using a weekly exercise log and physical activity questionnaire (21). Data collection occurred on two separate days, with an average of ~16 days between the study visits. Additionally, the MRI scan was conducted with the participants in a rested and fasted state, similar to the laboratory study day when cfPWV was measured. All study procedures were approved by the Institutional Review Board of the University of Wisconsin–Madison and were performed according to the Declaration of Helsinki, including obtaining written informed consent from each participant.

### Aortic Stiffness

To assess aortic stiffness, carotid-femoral pulse wave velocity (cfPWV) was measured using applanation tonometry (Sphygmocor, AtcorMedical, Sydney, NSW, Australia). High-fidelity pressure waveforms were measured at the common carotid and common femoral arteries as previously described (22). Briefly, a pencil like probe was placed on each of the arteries and pressure waveforms were recorded in order to determine the delay of the foot of the pressure waves from the R wave recorded using an electrocardiogram. The distance was calculated using the length between the common carotid and femoral arteries minus the distance from the suprasternal notch and the carotid pulsating point. This distance was divided by the time delay between the pressure waveforms to calculate cfPWV. An average of three measurements were used for the analysis (23). Additionally, using the same device, an aortic pressure waveform was derived from the radial pulse using the application of a generalized transfer function to measure augmentation index (AIx), which was corrected at a heart rate (HR) of 75 beats per minute. Aortic pulse pressure was calculated by subtracting aortic diastolic blood pressure from aortic systolic blood pressure. Augmentation pressure was also calculated during the pulse wave analysis as the height above diastolic pressure of the first shoulder of the aortic pressure waveform (24). We included AIx in our stepwise regression analyses to determine if it was a significant predictor for cerebral pulsatility, as it is an index of arterial wave reflection that is indirectly associated with arterial stiffness.

### Magnetic Resonance Imaging and Flow Analysis

Cranial MRI scans were performed at the Wisconsin Institutes for Medical Research using a 3T clinical MRI system (MR750, GE Healthcare, Waukesha, WI, United States) and a 32-channel head coil (Nova Medical Head Coil, Nova Medical, Wilmington, MA, United States) with a gradient strength of 50 mT/m, and a gradient slew rate of 200 mT/m/ms. Cerebral artery pulsatility index was assessed using 4D Flow Phase Contrast MRI using a 3D radially undersampled sequence that included volumetric, time-resolved PC MRI data with three-directional velocity encoding (PC-VIPR) (25, 26). The imaging parameters were as follows: velocity encoding (Venc) = 80 cm/s, field of view = 220 mm, acquired isotropic spatial resolution = 0.7 mm × 0.7 mm × 0.7 mm, repetition time (TR) = 7.4 ms, echo time (TE) = 2.7 ms, flip angle = 10°, bandwidth = 83.3 kHz, 14,000 projection angles and scan time ~7 min. Time-resolved velocity and magnitude data were reconstructed offline by retrospectively gating into 20 cardiac phases using temporal interpolation (27, 28).

The 4D Flow MRI scans were evaluated offline. The scans underwent background phase offset correction, eddy current correction (29) and automatic phase unwrapping to minimize potential for velocity aliasing (30). Vessel segmentation of right and left ICAs, MCAs, and basilar artery were performed in MATLAB using an in-house tool as previously described for semi-automated cerebrovascular hemodynamic analysis (29). The ICAs were measured below the carotid siphon along the cervical and petrous portions. The basilar artery was measured above the bifurcation of the vertebral arteries and below the superior cerebellar artery. The MCAs were measured at the M1 segment. Pulsatility index for each artery was calculated as (maximum flow – minimum flow) / mean flow.

### Statistical Analyses

Data analyses were performed using the Statistical Package for the Social Sciences version 28 (SPSS, IBM Corp., Armonk, NY, United States). Statistical differences in participant characteristics between young and older adults were evaluated using Student's *t*-tests for unpaired data. Associations of interest were analyzed by Pearson correlational analyses and stepwise regression analyses. The Fisher *r*-to-*z* transformation was used to compare correlations of interest. Additionally, multivariable linear regression was used to evaluate if age and cfPWV would be a significant predictor for cerebral pulsatility index. Statistical significance was set α priori at *p* < 0.05.

## Results

### Age Group Comparisons

Participant characteristics are presented in Table 1, while cerebral vessel data are presented in Table 2. There were no group differences between the young and older adults for height, weight, BMI, MET minutes per week, and resting HR. However, the older adults had elevated systolic blood pressure, diastolic blood pressure, mean arterial pressure, augmentation index, augmentation pressure, and cfPWV. Cerebral artery characteristics are shown on Table 2. As expected, pulsatility index was higher in the ICAs, MCAs, and basilar artery in the older adults compared with the young adults. Importantly, the diameter of the ICAs, MCAs, and basilar artery were equivalent between the young and older adults.

**Table 1:** Characteristics of participants.

**Variable**	**Young adults** ***N*** **= 33**	**Older adults** ***N*** **= 23**	***p*** **-value**
Males / Females (n)	16 / 17	13 / 10	
Age (years)	25 ± 4	62 ± 6	<0.001
Height (cm)	172 ± 7	174 ± 8	0.54
Weight (kg)	69 ± 9	73 ± 14	0.20
Body mass index (kg/m^2^)	23 ± 2	24 ± 3	0.18
Heart rate at rest (beats per minute)	55 ± 8	57 ± 7	0.38
MET minutes per week	3,076 ± 1,718	3,002 ± 2,141	0.27
Systolic blood pressure (mmHg)	119 ± 10	125 ± 10	0.04
Diastolic blood pressure (mmHg)	69 ± 6	76 ± 6	<0.001
Mean arterial pressure (mmHg)	86 ± 7	93 ± 7	0.001
AIx (%)	−0.8 ± 10.6	16.6 ± 9.1	<0.001
Augmentation pressure (mmHg)	2.6 ± 4.0	10.5 ± 5.3	<0.001
cfPWV (m/s)	6.2 ± 0.9	8.2 ± 1.5	<0.001

*MET, metabolic equivalent, AIx, augmentation index at 75 beats per minute, cfPWV, carotid-femoral pulse wave velocity. Data are presented as mean ± standard deviation*.

**Table 2:** Cerebral vessel variables.

**Variable**	**Young Adults** ***N*** **= 33**	**Older Adults** ***N*** **= 23**	***p*** **-value**
Internal carotid artery diameter (mm)	4.4 ± 0.3	4.5 ± 0.3	0.18
Internal carotid artery pulsatility index (a.u.)	0.92 ± 0.09	1.01 ± 0.10	0.001
Middle cerebral artery diameter (mm)	2.9 ± 0.2	2.9 ± 0.2	0.69
Middle cerebral artery pulsatility index (a.u.)	0.98 ± 0.11	1.08 ± 0.12	0.003
Basilar artery diameter (mm)	3.2 ± 0.2	3.1 ± 0.3	0.33
Basilar artery pulsatility index (a.u.)	0.96 ± 0.10	1.11 ± 0.16	<0.001

*Data are presented as mean ± standard deviation*.

### Correlation Analyses

**Figure 1:**
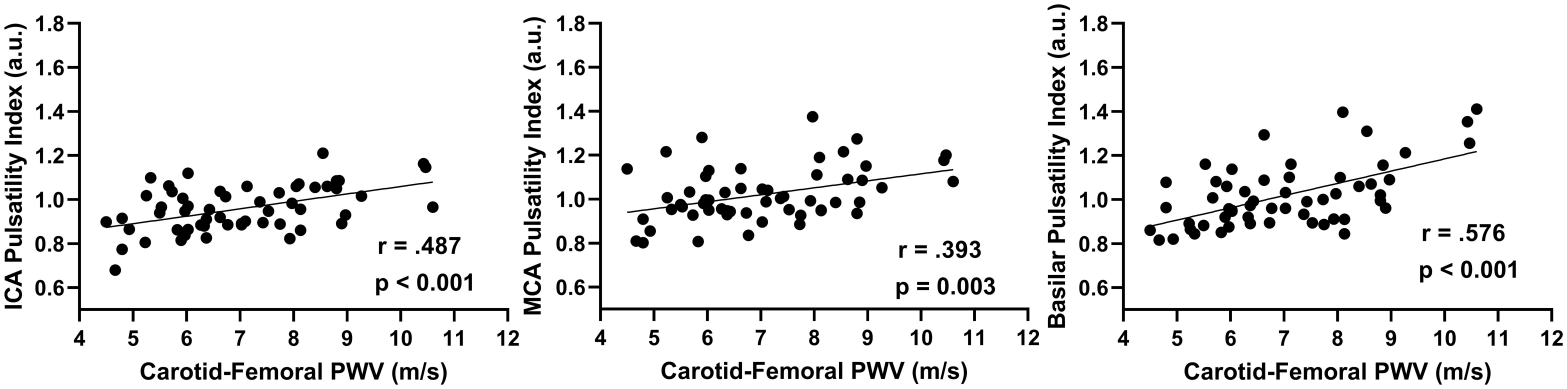
The relationship between internal carotid artery (ICAs), middle cerebral artery (MCAs), and basilar artery pulsatility index with aortic stiffness (cfPWV) in combined age groups (*N* = 56) using Pearson correlations.

**Figure 2:**
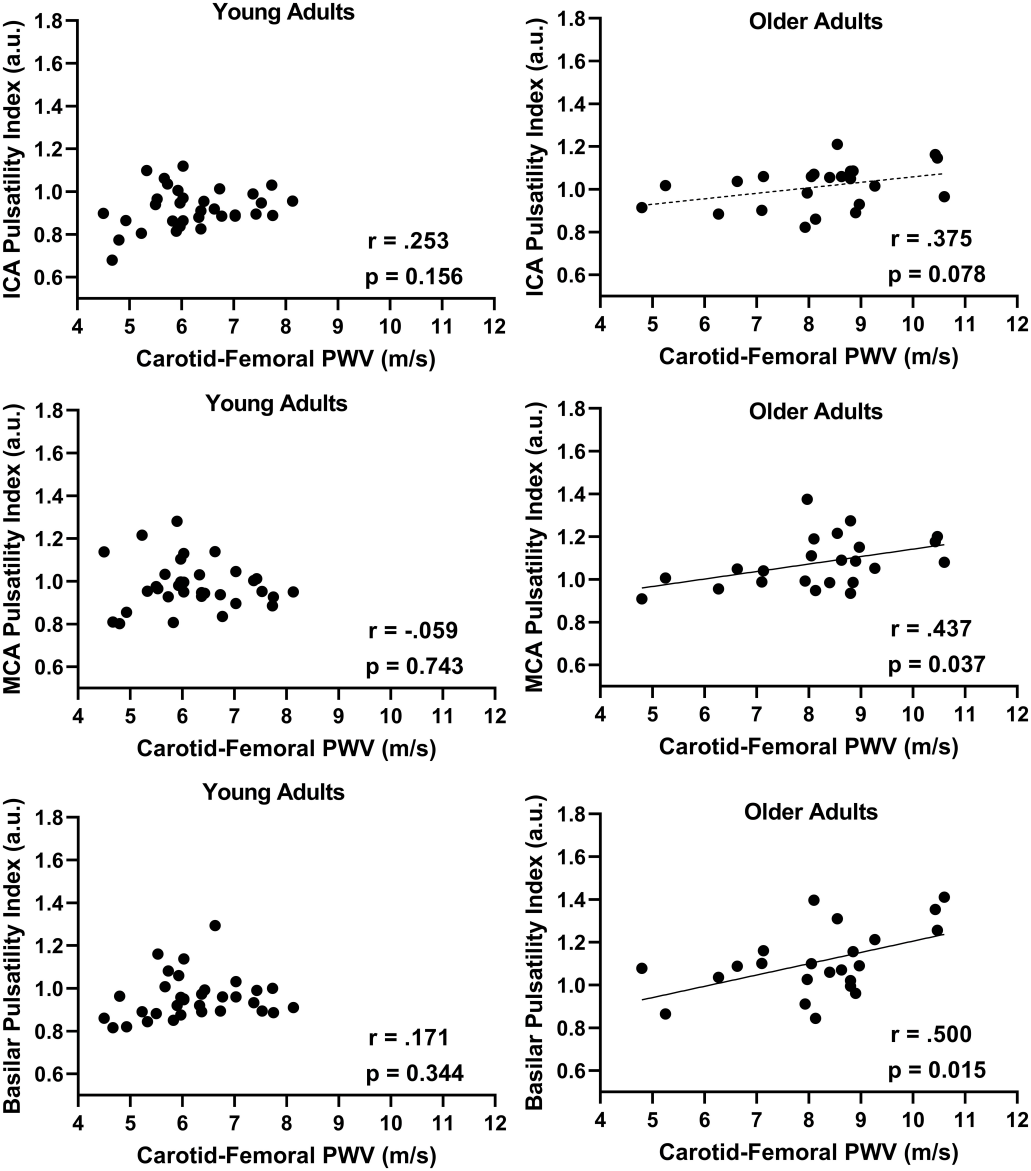
The relationship between internal carotid artery (ICAs), middle cerebral artery (MCAs), and basilar artery pulsatility index with aortic stiffness (cfPWV) split by age groups, young adults (*N* = 33) vs. older adults (*N* = 23) using Pearson correlations.

In the combined group, there was a positive association between aortic stiffness (cfPWV) and the cerebral artery pulsatility index for the averaged ICAs (*r* = 0.487; *p* < 0.001), averaged MCAs (*r* = 0.393; *p* = 0.003), and basilar artery (*r* = 0.576; *p* < 0.001) as shown in Figure 1. Additionally, when using partial correlations to determine the influence of age with cfPWV and pulsatility index, the association remained significant for the averaged ICAs (*r* = 0.277; *p* = 0.041) and basilar artery (*r* = 0.310; *p* = 0.021); however, the correlation for averaged MCAs was no longer significant (*r* = 0.200; *p* = 0.143) indicating this association is driven by age. Next, when evaluating the associations within each group (presented in Figure 2), there was no association between cfPWV and ICA pulsatility index in the young adults (*r* = 0.253; *p* = 0.156) and the association did not reach the threshold for significance in older adults (*r* = 0.375; *p* = 0.078). In addition, although there was no association between cfPWV and MCA pulsatility index in young adults (*r* = −0.059; *p* = 0.743), the association was significant in older adults (*r* = 0.437; *p* = 0.037). Similarly, there was no relationship between cfPWV and basilar artery pulsatility index in young adults (*r* = 0.171; *p* = 0.344), while there was a significant positive association in older adults (*r* = 0.500; *p* = 0.015).

We also investigated the associations of aortic pulse pressure and cerebral pulsatility index, in the combined age group aortic pulse pressure was associated with cerebral pulsatility index in the ICAs (*r* = 0.334, *p* = 0.014), MCAs (*r* = 0.312, *p* = 0.023), and basilar artery (*r* = 0.398, *p* = 0.003). Using the Fisher *r*-to-*z* transformation these correlations are not significantly different to those of cerebral pulsatility index and cfPWV for the ICAs (*z* = 0.95, *p* = 0.342), MCAs (*z* = 0.48, *p* = 0.631), and basilar artery (*z* = 1.21, *p* = 0.226). When evaluating the associations within each age group, there was no relationship between aortic pulse pressure and cerebral pulsatility index in young adults (ICAs: *r* = 0.191; *p* = 0.296, MCAs: *r* = 0.199; *p* = 0.275, basilar artery *r* = 0.317; *p* = 0.077). Similarly, there were no associations between aortic pulse pressure and cerebral pulsatility index in any of the arteries of interest in the older adults (ICAs: *r* = 0.230; *p* = 0.316, MCAs: *r* = 0.183; *p* = 0.427, basilar artery *r* = 0.188; *p* = 0.416).

We also investigated regional differences in pulsatility index of the left and right ICA and MCA for associations with cfPWV, as anatomical variations may influence results. Interestingly, in the older participants we found significant correlations for the left ICA (*r* = 0.418; *p* = 0.047) and left MCA (*r* = 0.440; *p* = 0.035) with cfPWV, but not for the right ICA (*r* = 0.306; *p* = 0.155) and right MCA (*r* = 0.227; *p* = 0.297). Similar to the averaged data, there were no significant correlations in either the left ICA (*r* = 0.218; *p* = 0.223) or right ICA (*r* = 0.266; *p* = 0.135) and left MCA (*r* = −0.224; *p* = 0.211) or right MCA (*r* = 0.258; *p* = 0.148) for the young participants.

### Regression Analyses

Stepwise regression analyses were used to determine the main predictive variables responsible for the observed associations. The stepwise regression analyses revealed that the strongest correlate for ICA pulsatility index was cfPWV, explaining 19% of the variance. For MCA pulsatility index, cfPWV explained 16% of the variance. Similarly, with basilar artery pulsatility index, cfPWV explained 30% of the variance. These results included all of the participants with the averaged ICAs and MCAs. We then used multiple linear regression with age and cfPWV to test our hypothesis that they would be significant predictors of cerebral pulsatility index. Indeed, there was a significant association for averaged ICA pulsatility index (β (SE) = 0.744 (0.062), *p* < 0.001), averaged MCA pulsatility index (β (SE) = 0.822 (0.077), *p* < 0.001), and basilar artery pulsatility index (β (SE) = 0.677 (0.079), *p* < 0.001) when all the participants were included. Additionally, age and cfPWV explained 26% of the variance for ICA pulsatility index, 17% for MCA pulsatility index, and 38% for basilar artery pulsatility index.

## Discussion

Our study is the first to demonstrate that aortic stiffness (cfPWV) is positively correlated with cerebral artery pulsatility index within the ICAs, MCAs, and basilar artery using 4D Flow MRI. Although we hypothesized that ICA pulsatility index would be associated with cfPWV in the older adults, this association did not reach the threshold for significance. The ICAs have a larger diameter than the MCAs and basilar artery and previous research has demonstrated that increased arterial diameter influences pulsatility index (31). As such, we speculate that the ICAs may have higher elasticity than the MCAs and basilar artery, which may account for the slightly lower pulsatility index values in the ICA compared to the MCAs and basilar artery. However, as expected, we did demonstrate a significant association between cfPWV and MCA pulsatility index in the older adults. The overall association between cfPWV and MCA pulsatility index with the combined age groups was dependent on age, as the partial correlation was no longer significant when controlling for aging. Further illustrating that with advancing age, cerebral pulsatility index increases with elevated aortic stiffness. Additionally, the multiple linear regression analysis supported the hypothesis that both age and cfPWV are significant predictors of cerebral vessel pulsatility index. As predicted, this relationship was only present in the older adults when we evaluated the age groups.

Our study is novel in that we utilized 4D Flow MRI to measure pulsatility in multiple cerebral vessels (ICAs, MCAs, and basilar artery) to comprehensively assess the effect of aortic stiffness on cerebral pulsatility with advancing age. Additionally, because the older adults included in this study were healthy, we were able to elucidate the role aging plays on the relationship between aortic stiffness and intracranial cerebral pulsatility. Importantly, our findings are in agreement with previous data that cfPWV is positively associated with MCA pulsatility index using transcranial doppler ultrasonography, which measures blood velocity (19), whereas the current study calculated pulsatility index using flow.

It is well-established that with advancing age, arterial stiffness increases (7, 32) and does not seem to be dependent on atherosclerosis (33). Over time, mechanical fraying of elastin structures occurs within the vessel wall from repeated bouts of mechanical stress and crosslinking of collagen fibers from advanced glycation end-products. This process leads to increased stiffening of the arteries (34–36). With elevated arterial stiffening, pulsatile flow within the arteries are generated via myocardial contraction, which propagates to the peripheral microvasculature and results in end organ damage (37). The brain is especially susceptible to excessive pulsatility due to its high demand for blood flow and low resistance of the arterioles located in the brain (38, 39). This highlights the importance of large conducting arteries to buffer pulsatility prior to reaching the cerebral circulation (40, 41). For example, pulsatility index in the ICA decreases from proximal to distal in the carotid siphon, aiding to attenuate pulsatility (42). Additionally, accelerated stiffening of the aorta with aging is directly related to end-organ damage to the brain via increased microvascular brain pulsatility and development of cerebral small vessel disease (43). Previous research has demonstrated aortic stiffness measured via phase-contrast MRI predicts white matter hyperintensities (44), while aortic stiffness measured with cfPWV is also associated with white matter hyperintensities and cognitive decline (45).

Arterial stiffness is a sensitive predictor of cognitive dysfunction and has been suggested to be a target to reduce or delay the onset and progression of dementia in older adults (46). Previous studies have suggested that aging-related increases in aortic stiffness results in impedance matching with stiffer peripheral muscular arteries, that results in reduced wave reflections at first-order bifurcations (10). However, other studies have suggested that impedances are relatively well-matched between central elastic arteries and peripheral muscular arteries prior to aortic stiffening observed with advancing age, although increased pulsatile energy from the central arteries can penetrate and damage the cerebral microvasculature (45). Our data supports the notion that increased aortic stiffness with aging results in elevated cerebral pulsatility. Furthermore, elevated arterial stiffness is associated with worse executive function, memory, and global cognition (47). As such, a recent meta-analysis concluded elevated arterial stiffness with older adults is associated with deteriorating memory and processing speed (48) and higher aortic stiffness is associated with faster cognitive decline (49). Our study attempts to provide a mechanistic link (i.e., cerebral pulsatility) to help explain why increases in aortic stiffness with advancing age in humans may result in cognitive decline.

In the present study, older adults had higher pulsatility index in the cerebral vasculature compared with young adults, which was associated with elevated aortic stiffness. As such, we speculate that this age-related increase in aortic stiffness plays a role in pulse wave encephalopathy (50). This term describes the process of how increased pulsatile flow results in microvascular damage in the brain and increases the risk for mild cognitive impairment and dementia (38, 51). Therefore, reducing aortic stiffness at mid-life may be successful in preventing or delaying the onset of stroke, dementia, and other consequences of cerebral small vessel disease (52). Recently, it has been demonstrated that increased estimated cardiorespiratory fitness was associated with decreased pulsatility index in large cerebral vessels (28). Moreover, a “normal” heart rate (around 75 bpm) has been demonstrated to provide an optimum wave condition in the aorta to attenuate cerebral pulsatility (53). These findings further highlight the need to develop interventions (e.g., exercise) to mitigate age-related increases in aortic stiffness to help reduce the risk of developing cognitive dysfunction with our growing aging population.

This study is not without limitations. First, a larger sample size may be needed to demonstrate a significant correlation between with cfPWV and ICAs pulsatility index in older adult cohorts. Second, this study used a cross-sectional design to infer the impact of aging on associations of aortic stiffness and cerebral pulsatility index. Third, although there is a strong correlation between tonometry-based cfPWV and PC-MRI aortic PWV values, error is introduced by the use of linear surface measurements for length to determine carotid to femoral distance (54). However, this error could be considered negligible because age-related aortic stiffness is mainly influenced by decreased pulse transition time (55). Finally, the older adults included in this study were healthy, which may limit the translation of this study to the general population who typically have cardiovascular risk factors or disease. Future longitudinal studies are needed to confirm these relationships.

In conclusion, our study demonstrated that age and cfPWV are significant predictors of cerebral pulsatility index (ICAs, MCAs, and basilar artery). Additionally, with combined age groups, arterial stiffness is associated with pulsatility index in each of the cerebral vessels examined. These relationships remained significant in the older adults for the MCAs and basilar artery. This study highlights the importance of targeting aortic stiffness in our increasingly aging population to reduce the burden of age-related changes in cerebral hemodynamics.

## Data Availability Statement

The raw data supporting the conclusions of this article will be made available by the authors, without undue reservation.

## Ethics Statement

The studies involving human participants were reviewed and approved by Institutional Review Board of the University of Wisconsin–Madison. The patients/participants provided their written informed consent to participate in this study.

## Author Contributions

KM and JB designed the experiments. KM, AC, AH, NE, AP, and JB collected the data. BF, KM, AH, LR-R, HR, and JB analyzed the data. BF, KM, LR-R, OW, KJ, SJ, and JB interpreted the data. BF, KM, and JB wrote the manuscript and prepared figures. All authors edited, revised, and approved the final version of the manuscript.

## Funding

This study was supported by the National Institutes of Health (R00HL118154 to JB). This investigation was also supported by the National Institutes of Health, Ruth L. Kirschstein National Research Service Award T32's from the under National Institute on Aging to the University of Wisconsin–Madison Biology of Aging & Age-Related Diseases (AG000213 to BF) and under the National Heart Lung and Blood Institute to the University of Wisconsin–Madison Cardiovascular Research Center (HL007936 to KM) as well as the Wisconsin Alumni Research Foundation (JB). We would like to thank the Marsh Center for Exercise and Movement Research for the generous support of this work.

## Conflict of Interest

The authors declare that the research was conducted in the absence of any commercial or financial relationships that could be construed as a potential conflict of interest.

## Publisher's Note

All claims expressed in this article are solely those of the authors and do not necessarily represent those of their affiliated organizations, or those of the publisher, the editors and the reviewers. Any product that may be evaluated in this article, or claim that may be made by its manufacturer, is not guaranteed or endorsed by the publisher.
